# Factors affecting provision of female family planning commodities in public health facilities in Kajiado county, Kenya

**DOI:** 10.1186/s40545-022-00488-y

**Published:** 2022-11-24

**Authors:** Fredrick Githinji, Shital M. Maru, Peter N. Karimi, Eugene Rutungwa, Egide Kayitare

**Affiliations:** 1 EAC Regional Centre of Excellence for Vaccines, Immunization, and Health Supply Chain Management, College of Medicines and Health Sciences, University of Rwanda, Kigali, Rwanda; 2 Faculty of Health Sciences, University of Nairobi, Nairobi, Kenya; 3 School of Business, College of Business and Economics, University of Rwanda, Kigali, Rwanda; 4 School of Medicine and Pharmacy, College of Medicine and Health Sciences, University of Rwanda, Kigali, Rwanda

**Keywords:** Provision, Family planning, Public facilities, Kajiado county, Kenya

## Abstract

**Background:**

Family planning involves the use of traditional or modern methods to prevent maternal and infant mortality associated with unintended pregnancies and negative economic outcomes. In sub-Saharan Africa, the unmet need for modern family planning is approximately 66%. However, information on factors affecting utilization of female family planning commodities is limited. Therefore, this research was conducted to bridge this gap.

**Methods:**

Health facility-based descriptive cross-sectional research design was conducted and involved the public health facilities offering family planning, targeting respondents who handle the commodities and service providers themselves. A semi-structured questionnaire was used to collect data about availability of the commodities, knowledge of service providers and barriers affecting provision of the service. Data were coded and analyzed via Microsoft Excel 2019 and SPSS version 20.

**Results:**

The study showed that shorter term methods were more readily available, 60–75% than the long-term methods, 20–60%. Approximately 60% of the service providers did not comprehensively utilize the recommended World Health Organization Medicine Eligibility Criteria (WHO MEC) during service provision. Stock outs, myths and misconceptions, male interference and culture were the major barriers identified.

**Conclusion:**

Provision of family planning commodities in public health facilities in Kajiado county is affected by stock levels at the national program, and provider knowledge on WHO MEC. The key factors affecting provision of family planning were stock outs, myths and misconceptions on the contraceptives, inadequate male involvement and inadequate community engagement on potential benefits of the service. These challenges need to be part of the solutions to bridging the gap identified.

## Background

Family planning involves the use of traditional or modern methods. Modern methods incorporate the use of short or long-term methods, depending on the classification by a program or country, whose length of preventing or delaying a pregnancy is measured in time [1]. Effective use of modern family planning methods saves lives through prevention of maternal and infant mortality associated with unintended pregnancies [2]. It also fosters better economic growth and reduces the dependency ratio of a country’s population [3].

Globally, utilization of modern family planning methods increased from 42 to 49%, after the International Conference on Population and Development at Cairo, Egypt in 1994, with varying fertility rates within and across regions [3]. High-income countries have the lowest fertility rates, while low-income countries like those in sub-Saharan Africa have the highest fertility rates [4].

In sub-Saharan Africa, the unmet need for modern family planning is approximately 66%, and affects the slow reduction in fertility rates compared to other regions [5]. Other factors such as socio-demographic characteristics, structure of the health systems, fear of side effects, education levels and other barriers, contribute further to the limited access as a challenge to provision of family planning methods in the region [6].

In East Africa, the contraceptive prevalence rate varies across countries, with Rwanda having 64% of married women utilizing any method [7] within a span of 10 years. The change was less drastic in Uganda at 30% [8] and Tanzania at 32% [9], having stagnated in the early twenty-first century [10]. A study conducted in South Sudan, placed the number of women on any form of contraceptive method at 4.7% [11]. However, in Burundi contraceptive prevalence as at 2017 was at 23.8% [12]. Despite Kenya having had contraceptive prevalence of 58%, this change was more gradual between 1993 and 2014 [13].

The most recent demographic health survey in Kenya of 2014/15, reported that the contraceptive prevalence rate was at 58% [14]. Inadequacy in resource allocation, forecasting, provision of long-term or permanent methods and weak inventory management practices, are among the major concerns in utilization of family planning commodities within the country [15]. The national family planning program carries out annual forecasts for the required supplies through its partners and utilizes data reported from the counties in conducting the process [16]. A recent assessment conducted in 2015, showed that delayed replenishment of commodities was the main cause for the stock outs, followed by inadequacy of trained staff that contributed to low service availability [17]. In Kenya, national referral hospitals, county and private health facilities serve as the service delivery points where family planning services are offered alongside inventory management of the accompanying supplies.

In Kajiado county, the current contraceptive prevalence rate is at of 45.2% [14] against the national rate of 58%. Low contraceptive prevalence rate (CPR) translates to unmet need with the potential of increasing maternal, infant and child mortality rates, reduced quality of lives for mothers, increase in teenage pregnancies and slow economic development [18]. A study conducted in East and Southern Africa concluded that stock outs of family planning methods in public sector may lead to further challenges of inaccessibility by clients especially where the alternative is seeking them in the private sector where affordability becomes a compounding challenge [19].

Information on factors affecting provision of female family planning commodities in public health facilities at counties within Kenya is limited. Therefore, this research was conducted to identify factors affecting provision of female family planning commodities.

## Methods

### Study area and period

Data were collected from October to December 2021 within selected public health facilities in Kajiado county, located in the southern part of Kenya. It covers an area of approximately 21,900 km^2^. Administratively, it is subdivided into 5 sub counties with an approximate population of 1,117,840 people as per the 2019 census and a total of 110 public facilities offering family planning services.

### Sampling design

The study adopted a descriptive cross-sectional design using semi-structured questionnaires to capture the required data.

### Study population and sampling

The study population comprised 86 health care workers responsible for family planning commodities in the pharmacy and 85 family planning service providers. The sample size was 86 facilities which were obtained using Yamane’s formula that provides a 95% confidence interval. These facilities were stratified as per the Kenya essential package of health (KEPH). They included dispensaries, health centers, sub county hospitals and referral hospitals. For the female family planning commodities, six products were assessed in each health facility; combined oral contraceptive pills (COCs), progestin only pills (POPs), deoxy medroxyprogesterone acetate (DMPA) injection, etonogestrel implants, levonorgestrel implants and intra-uterine contraceptive devices (IUCDs). All the hospitals were sampled, due to the few numbers in that KEPH level. For the health centers and dispensaries, systematic sampling was done guided by proportions in each tier.

### Data collection tools

Semi-structured questionnaire was used to collect the data. It had several variables categorized as biodata, types of health commodities and duration of stock outs. To assess knowledge, the WHO MEC was used to assess the key steps of family planning provision which also incorporated any challenges that they may be facing as well. The questionnaire was pre-tested and validated to check for reliability before conducting the actual data collection.

### Data collection procedures

Research assistants were trained on how to how to ask questions and fill the questionnaire. This was followed by pre-testing of the tool where respondents from nine facilities drawn from the pharmacy, and family planning clinics were involved. Adjustments were done on the questionnaire to make it more comprehensive. The principal investigator printed the revised questionnaire and sent it to the consenting respondents who filled it as appropriate. A physical site visit was then conducted by the researcher and assistants, to access the bin cards for purposes of assessing the stock status and getting the challenges faced during service provision by the respondents. The filled questionnaires were collected concurrently for further processing.

### Data management, analysis and quality assurance

Quantitative data were entered, cleaned, and coded using Microsoft excel 2019 prior to the analysis. Descriptive statistics were used to analyze data and data were presented using frequency and percentage using the SPSS version 20.

## Results

### Socio-demographic characteristics of the study respondents

The family planning commodities were being handled by different cadres of health care providers (Table 1). Majority were nurses 57 (67.86%), who were performing additional duties, while pharmacists/pharmaceutical technologists and clinical officers were the least. Around half of the participants were male 47 (55.95%) and the majority of the participants 65 (77.38%) had a diploma.

**Table 1:** Demographic characteristics of health workers family planning commodities

Characteristic	Variable	Number	Percentage
Job title	Pharmacist/Pharm tech	14	16.67
	Nurse	57	67.86
	Clinical officer	12	14.29
	Other	1	1.19
Gender	Male	47	55.95
	Female	37	44.05
Age	20–29	19	22.62
	30–39	48	57.14
	40–49	17	20.24
Level of Education	Post graduate	3	3.57
	Degree	5	5.95
	Diploma	65	77.38
	Certificate	11	13.10
Department	Pharmacy	15	17.86
	Stores	2	2.38
	Other	67	79.76

### Availability of female family planning commodities

**Fig. 1:**
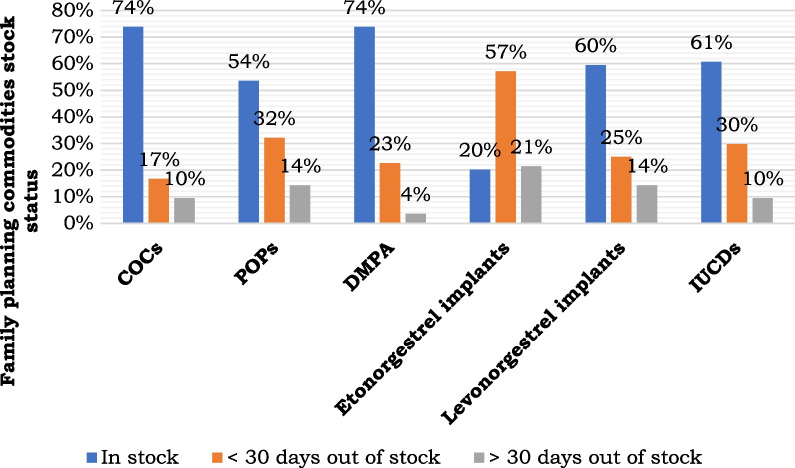
Stock status of female planning commodities. *COCs*  combined oral contraceptives, *POPs*  progestin only pills, *DMPA* deoxy medroxy progesterone acetate, *IUCDs* intra-uterine contraceptive devices

The products that were readily available are shown in Fig. 1. The stock out period was longer for the implants, up to 57% followed by POPs with 32%, IUCDs up to 30%, while the DMPA had 23% compared to COCs which had 17% stock out within 30 days.

### Knowledge of staff on rational provision of female planning commodities

For service provision as per WHO MEC criteria, 76.47% of respondents asked clients to mention any family planning methods they knew of as part of the process and 89.41% gave a return date. However, only 56.47% informed their clients on any other modern FP methods they may not know of, while 57.65% went further into educating clients on advantages, disadvantages and any side effects of the informed method of choice (Table 2).

**Table 2:** Family planning services offered according to WHO MEC

Type of service provided	*n* (%)
Takes client history and conducts a physical exam	57 (67.06)
Asks client to mention any FP methods they know	65 (76.47)
Informs client about any other FP methods not mentioned	48 (56.47)
Assists the client make an informed choice as per WHO MEC	54 (63.53)
Informs the client on advantages, disadvantages and any side effects	49 (57.65)
Counsel the client on how to administer the method	59 (69.41)
Give a return date	76 (89.41)

### Barriers to provision of female FP commodities

**Fig. 2:**
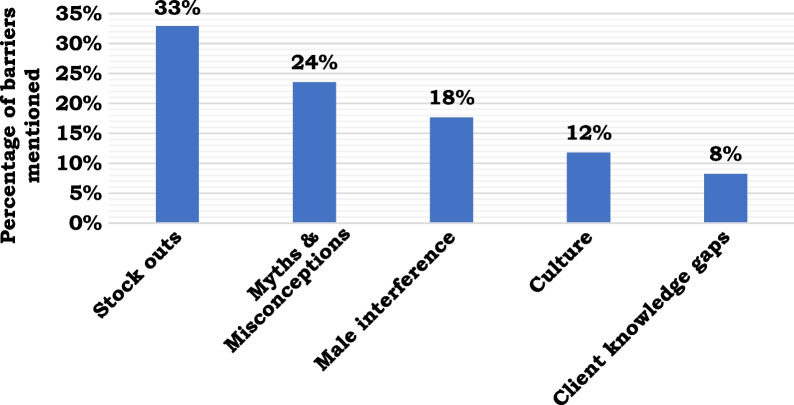
Barriers to provision of female family planning commodities

Stock outs (33%), myths and misconceptions (24%), male interference (18%) and culture (12%) were the major barriers for the provision of female family planning commodities. Figure 2 provides more details.

## Discussion

The purpose of this study was to find out the factors that affect provision of female family planning commodities in public health facilities in Kajiado county, Kenya.

### Availability and stock out

Short-term methods were more readily available than the long-term methods. Stock outs affect the health seeking behavior of clients who may need the service and may cause missed opportunities that are due to the health system not being adequately responsive. This finding corroborates a cross country research conducted in Kenya, Tanzania, Uganda and Zambia [19]. Stock outs may lead to clients seeking services in private sector where cost may be another challenge. An assessment conducted in ten African countries also demonstrated the varying availability of some methods for contraception [8]. Stock outs of supportive equipment and other logistical supplies may affect the quality-of-service provision. It is therefore important to have all requisite supplies required to offer the family planning services in totality.

### Knowledge of service providers

Improvised forms of service provision or even turning away clients may be a consequence of inadequacy in skills [20]. Provider knowledge may also influence the family planning method which a client will receive. In this study, almost half of the respondents did not inform clients of methods they may not be aware of or even the benefits or risks of the method they chose after being informed. This in itself is a gap, because clients are limited in making informed decisions as part of the counselling offered to assist in selecting an appropriate method. In relation to this, provider imposed bias may be beyond medical reasons and more geared towards personal and self-imposed purposes [21]. The findings of this study may be of significance in that, the providers may be a hindrance to the clients in ensuring they get the best quality care that is of benefit to them. Additionally, compromising the quality in turn may be of a disadvantage to the clients as evidenced in another study [22].

### Barriers to provision of family planning commodities

In addition to stock outs, myths and misconceptions, male interference and culture were found to be other contributing factors affecting provision and subsequent utilization of family planning commodities. In Nigeria [18] a study revealed the influence of spousal approval in seeking family planning by the women interviewed. Even though this study did not interview the clients, service providers working especially in rural areas reported this to be a major contributing factor to women seeking the service. This also led to women discreetly seeking the short-term injectable method, as a way of hiding it from their non-consenting spouses or community, for fear of being shunned away if discovered. This behavior was also tied to the cultural beliefs that modern family planning methods could lead to infertility. A study conducted in Kenya [23] also agreed with such findings and went further to state that, family planning was a way of allowing women to become promiscuous and may lead to side effects contributing to infertility.

### Limitations of the study

The target population was derived from the public sector. There are other sector players offering family planning, including the private for profit, not for profit and faith-based organizations. These players conduct joint community outreaches and have their own complementary supply chain that may give a different picture when investigated.

The cross-sectional research design also provided a snapshot of the situation which may not be the case if conducted at a different point in time. Lastly, the study focused on the service provider and did not include the women of reproductive age who seek the commodities and service. Future studies that target clients are encouraged.

## Conclusion

The current study aimed to determine factors that affect provision of female family planning commodities in public health facilities in Kajiado county, Kenya. The findings revealed that provision of family planning commodities in public health facilities in Kajiado county is affected by stock levels at the national program and provider knowledge/skills gaps in provision of modern methods. The key challenges are stock outs, myths and misconceptions on the various methods, inadequate male involvement and inadequate community engagement on potential benefits of the service.

## Data Availability

The data used to produce the current manuscript are available upon a reasonable request to the corresponding author.

## Abbreviations

AMC: Average monthly consumption
CHVs: Community health volunteers
COCs: Combined oral contraceptives
CPR: Contraceptive prevalence rate
CYP: Couple years of protection
DMPA: Deoxy medroxy progesterone acetate
FCDRR Tool: Facility consumption data report and request tool
FP: Family planning
FP CIP: Family planning costed implementation plan
IUCDs: Intra-uterine contraceptive devices
IUS: Intra-uterine systems
KEMSA: Kenya Medical Supplies Authority
KEPH Policy: Kenya Essential Package for Health Policy (2014–2030)
KHIS: Kenya Health Information System
KNH/UON-ERC: Kenya National Hospital/University of Nairobi/Ethics and Research Committee
LNG-IUS: Levonorgestrel-releasing intrauterine system
MCH: Mother and child health
MOS: Months of stock
NACOSTI: National Commission for Science Technology & Innovation
NCPD: National Council for Population Development
NET-EN: Norethisterone
POPs: Progestin only pills
SPSS: Statistical Package for the Social Sciences
SWOT: Strength, Weaknesses, Opportunities, Threats
TFR: Total fertility rate
UNFPA: United Nations Population Fund
WHO MEC: World Health Organization Medical Eligibility Criteria

## References

[1] Festin MPR, Kiarie J, Solo J, Spieler J, Malarcher S, Van Look PFA (2016). Moving towards the goals of FP2020—classifying contraceptives. Contraception.

[2] Starbird E, Norton M, Marcus R (2016). Investing in family planning: key to achieving the sustainable development goals. Glob Health Sci Pract.

[3] World fertility and family planning 2020: highlights. World Fertil. Fam. Plan. 2020 Highlights. 2020. https://www.un.org/en/development/desa/population/publications/pdf/family/World_Fertility_and_Family_Planning_2020_Highlights.pdf.

[4] Alkema L, Kantorova V, Menozzi C, Biddlecom A (2013). National, regional, and global rates and trends in contraceptive prevalence and unmet need for family planning between 1990 and 2015: a systematic and comprehensive analysis. Lancet.

[5] Bongaarts J (2020). Trends in fertility and fertility preferences in sub-Saharan Africa: the roles of education and family planning programs. Genus.

[6] Lutalo T, Gray R, Santelli J, Guwatudde D, Brahmbhatt H, Mathur S (2018). Unfulfilled need for contraception among women with unmet need but with the intention to use contraception in Rakai, Uganda: a longitudinal study. BMC Womens Health.

[7] Survey H. National institute of statistics of Rwanda. Rwanda Demographic and Health Survey. 2019.

[8] Kabagenyi A, Reid A, Ntozi J, Atuyambe L. Socio-cultural inhibitors to use of modern contraceptive techniques in rural Uganda: a qualitative study. Pan Afr Med J. 2016;25. http://www.panafrican-med-journal.com/content/article/25/78/full/. 10.11604/pamj.2016.25.78.6613 PMC5324155 28292041

[9] Yussuf MH, Elewonibi BR, Rwabilimbo MM, Mboya IB, Mahande MJ (2020). Trends and predictors of changes in modern contraceptive use among women aged 15–49 years in Tanzania from 2004–2016: evidence from Tanzania Demographic and Health Surveys. PLoS ONE.

[10] Otieno V, Agwanda Otieno A, Khasakhala A. Trends in fertility preference implementation among selected Eastern African countries. F1000Research. 2020;9. 10.12688/f1000research.22064.1 PMC9020534 35465061

[11] Kane S, Kok M, Rial M, Matere A, Dieleman M, Broerse JE (2016). Social norms and family planning decisions in South Sudan. BMC Public Health BMC Public Health.

[12] Nkunzimana E, Sufiyan Babale M, Ndoreraho A, Nyandwi J (2021). Uptake of modern contraceptive methods among Burundian women and associated factors: analysis of demographic and health survey data, Burundi 2016–2017. East Afr Health Res J.

[13] Izugbara CO, Wekesah FM, Tilahun T, Amo-Adjei J, Tsala Dimbuene ZT. Family planning in east Africa: trends and dynamics. African Popul Health Res Cent. 2018;

[14] Ministry of Health. Kenya demographics health survey 2014. https://www.dhsprogram.com/publications/publication-fr308-dhs-final-reports.cfm.

[15] UNFPA Kenya. Family planning. https://kenya.unfpa.org/en/topics/family-planning-1.

[16] Ministry of Health K. National guidelines for quantification , procurement and pipeline monitoring for family planning commodities in Kenya. 2016.

[17] NCPD. Kenya health facility assessment report 2015. 2016.

[18] Nwachukwu I, Obasi OO (2008). Use of modern birth control methods among rural communities in Imo State, Nigeria. Afr J Reprod Health.

[19] Ooms GI, Kibira D, Reed T, Van Den Ham HA, Mantel-Teeuwisse AK, Buckland-Merrett G (2020). Access to sexual and reproductive health commodities in East and Southern Africa: a cross-country comparison of availability, affordability and stock outs in Kenya, Tanzania, Uganda and Zambia. BMC Public Health BMC Public Health.

[20] Zuniga C, Wollum A, Grindlay K, Douglas-Durham E, Higgins S, Barr-Walker J (2022). The impacts of contraceptive stock outs on users, providers, and facilities: a systematic literature review. Glob Public Health.

[21] Solo J, Festin M (2019). Provider bias in family planning services: a review of its meaning and manifestations. Glob Health Sci Pract.

[22] Tessema GA, Laurence CO, Mahmood MA, Gomersall JS. Factors determining quality of care in family planning services in Africa. JBI Database Syst Rev Implement Rep. 2016;14:103–14. http://journals.lww.com/01938924-201608000-00013 10.11124/JBISRIR-2016-003056 27635750

[23] Ochako R, Mbondo M, Aloo S, Kaimenyi S, Thompson R, Temmerman M (2015). Barriers to modern contraceptive methods uptake among young women in Kenya: a qualitative study. BMC Public Health.

